# New insights of engineered extracellular vesicles as promising therapeutic systems

**DOI:** 10.20517/evcna.2023.22

**Published:** 2023-04-27

**Authors:** Cheng Jiang, Hongxing Liu, Yuhui Liao, Yanyan Jiang

**Affiliations:** ^1^School of Medicine, The Chinese University of Hong Kong, Shenzhen 518172, Guangdong, China.; ^2^The Chinese University of Hong Kong, Shenzhen Futian Biomedical Innovation R&D Center, Shenzhen 518036, Guangdong, China.; ^3^The First Affiliated Hospital of Guangzhou Medical University, Guangzhou Medical University, Guangzhou 510120, Guangdong, China.; ^4^Molecular Diagnosis and Treatment Center for Infectious Diseases, Dermatology Hospital, Southern Medical University, Guangzhou 510515, Guangdong, China.; ^5^School of Materials Science and Engineering, Shandong University, Jinan 250061, Shandong, China.

**Keywords:** Extracellular vesicles, drug delivery, nanomedicine, cell-free therapy, engineering strategies

## Abstract

Extracellular vesicles (EVs) are natural biological particles that carry and deliver molecular fingerprints from parental cells to receptor cells, where they take effect. EVs are widely recognized for their role as intercellular communication mediators and high correlation with disease evolution, making them a valuable target in many aspects, especially biomarker profiling and therapeutics. In the past decade, scientists from various disciplines, including biology, physics, chemistry, materials science, electrical engineering, and mechanical engineering, have jointly devoted efforts to advance the study of EVs from fundamental molecular mechanisms to EV-based translational medicine, covering EV marker-based diagnostics and EV-based drug delivery. Diverse interfacial engineering strategies have been developed to facilitate *in vitro* and *in vivo* studies of EVs. This special issue, titled “Interfacial Engineering Strategies for EV *in vitro* and *in vivo* Studies”, focuses on understanding the engineering logic and design rules of EVs in biomedical fields, highlighting their therapeutic potential in combating many diseases. This will provide new insights into the construction of promising diagnostic and therapeutic systems.

## INTRODUCTION

Extracellular vesicles (EVs) are small, membrane-bound particles that are secreted by cells and can carry a range of biomolecules, including proteins, RNA, metabolites, *etc*. In recent years, studies on EVs have garnered significant interest due to their crucial role in intercellular communication and their potential as diagnostic and therapeutic tools^[1]^. Significant progress has been made in understanding EV biogenesis, cargo sorting, and uptake by recipient cells. Additionally, recent studies have highlighted the potential of engineered EVs in various disease contexts, such as cancer, cardiovascular disease, and neurodegenerative disorders^[2-4]^. Novel techniques for isolating and characterizing EVs continue to be developed, and ongoing research is aimed at improving our understanding of EV biology and exploiting EVs as biomedical tools^[5-7]^. The special issue of “Interfacial Engineering Strategies for EV *in vitro* and *in vivo* Studies” aims to provide insights into the engineering logic and design rules of EVs by proper convergence of EV intrinsic physiochemical properties and the “add-on” functional ligands.

## ARTICLE DESCRIPTION

In the present special issue, a total of five review articles have been critically examined and published. The first review published by Liu *et al*. (2022) discussed the challenges of EVs as a nano-theranostic system and proposed a solution for nucleic acid functionalization based on recent advances^[8]^. Then, the diverse strategies of nucleic acid-functionalized EVs are summarized and the latest progress of nucleic acid-functionalized EVs in nanomedicine is highlighted. Finally, the challenges and prospects of nucleic acid-functionalized EVs as a promising diagnostic system are proposed.

Another review article from Liu *et al*. (2022) focuses on mammalian EVs (MEVs) and bacterial EVs (BEVs), which are the two most common types of EVs in the biomedical field^[9]^. In this review, the authors describe engineered MEVs and BEVs as promising nanocarriers for targeted therapy and summarize the biogenesis, isolation, and characterization of MEVs and BEVs. Then they describe engineering techniques for enhancement of the targeting ability of EVs. This review will help improve the understanding of engineered MEVs and BEVs, thereby promoting their application and clinical translation.

Liu *et al*. (2022) performed a critical review aimed at assessing the potential approaches for EV production and functional modification, and systematically summarizing the worldwide clinical trials initiated for various physiological systems with a specific focus on the biochemical effects of Mesenchymal stromal cells derived EVs (MSC-EVs) toward the therapy of eye diseases^[10]^. Recent advances in the therapy of ocular diseases based on MSC-EVs are demonstrated, and the associated challenges and prospects are discussed as well.

Shinge *et al*. (2022) presented an overview of the identified roles of plant exosome-like nanovesicles (PELNVs) in physiology and pathology^[11]^. They also provided new insight toward the engineering of the plant-originated EV for effective therapeutics besides the bacteria and mammalian cell, pointing out a clue for future direction to the ongoing research gaps.

The final review article by Aafreen *et al.* (2023) introduces recent efforts and knowledge advances in EV-based therapies^[12]^. They also outline currently available labeling strategies by which EVs can be combined with various imaging agents and/or therapeutic drugs and genes. A comprehensive review of prevailing EV imaging technologies is then presented along with examples and applications, with emphasis on imaging probes and agents, corresponding labeling methods, and the pros and cons of each imaging modality. Finally, the potential of theranostic EVs as a powerful new weapon in the arsenal of regenerative medicine and nanomedicine is summarized and envisioned.

Altogether, this special issue provides a comprehensive picture of the current situation and trends for the engineered EVs derived therapeutic systems, i.e. infant stage but bright future. Excitingly, a couple of companies have already been competing and chasing in the EV therapeutics market with proposed conceptional products, and we will witness more advances.

## DECLARATIONS

### Copyright

@ The Author(s) 2023.
